# Sphingosine-1-Phosphate-Triggered Expression of Cathelicidin LL-37 Promotes the Growth of Human Bladder Cancer Cells

**DOI:** 10.3390/ijms23137443

**Published:** 2022-07-04

**Authors:** Tomasz Wollny, Urszula Wnorowska, Ewelina Piktel, Łukasz Suprewicz, Grzegorz Król, Katarzyna Głuszek, Stanisław Góźdź, Janusz Kopczyński, Robert Bucki

**Affiliations:** 1 Holy Cross Oncology Center of Kielce, Artwińskiego 3, 25-734 Kielce, Poland; tomwollny@gmail.com (T.W.); kasia.kielce@wp.pl (K.G.); stanislawgozdz1@gmail.com (S.G.); janusz.kopczynski@onkol.kielce.pl (J.K.); 2 Department of Medical Microbiology and Nanobiomedical Engineering, Medical University of Bialystok, Mickiewicza 2C, 15-222 Bialystok, Poland; u.wnorowska@gmail.com (U.W.); lukaszsuprewicz@gmail.com (Ł.S.); 3 Independent Laboratory of Nanomedicine, Medical University of Bialystok, Mickiewicza 2B, 15-222 Bialystok, Poland; ewelina.piktel@wp.pl; 4 Department of Microbiology and Immunology, Institute of Medical Science, Collegium Medicum, Jan Kochanowski University of Kielce, IX Wieków Kielc 19A, 25-317 Kielce, Poland; g.krol@op.pl

**Keywords:** cancer, cathelicidin, bladder cancer, LL-37 peptide, sphingosine-1-phosphate

## Abstract

It has been proven that tumour growth and progression are regulated by a variety of mediators released during the inflammatory process preceding the tumour appearance, but the role of inflammation in the development of bladder cancer is ambiguous. This study was designed around the hypothesis that sphingosine-1-phosphate (S1P), as a regulator of several cellular processes important in both inflammation and cancer development, may exert some of the pro-tumorigenic effects indirectly due to its ability to regulate the expression of human cathelicidin (hCAP-18). LL-37 peptide released from hCAP-18 is involved in the development of various types of cancer in humans, especially those associated with infections. Using immunohistological staining, we showed high expression of hCAP-18/LL-37 and sphingosine kinase 1 (the enzyme that forms S1P from sphingosine) in human bladder cancer cells. In a cell culture model, S1P was able to stimulate the expression and release of hCAP-18/LL-37 from human bladder cells, and the addition of LL-37 peptide dose-dependently increased their proliferation. Additionally, the effect of S1P on LL-37 release was inhibited in the presence of FTY720P, a synthetic immunosuppressant that blocks S1P receptors. Together, this study presents the possibility of paracrine relation in which LL-37 production following cell stimulation by S1P promotes the development and growth of bladder cancer.

## 1. Introduction

Sphingosine-1-phosphate (S1P) participates in many cellular processes, acting as receptors’ agonists in cell survival, proliferation, migration, and angiogenesis [1,2]. It is produced from intracellular ceramide, which is initially converted into sphingosine by the enzyme ceramidase. In the next stage, sphingosine is phosphorylated by one of two sphingosine kinases (SphK) to form S1P [2]. Activation of one of five types of G-protein-coupled S1P receptors (S1PR1-5) can set in motion different signalling pathways [3] that regulate lymphocyte trafficking and maintenance of vascular integrity, thus contributing to the development of many inflammatory processes [4]. Moreover, a growing amount of evidence shows the role of S1P signalling in cancer; its importance has been described in tumour cell growth, apoptosis, progression of the disease and tumour metastasis, and chemotherapy resistance [5,6]. Therefore, it is believed that blockage of the S1P-S1PR signalling system could be a new therapeutic strategy for both inflammatory and oncological diseases. Notably, an active form of fingolimod (fingolimod-phosphate: FTY720P), a structural analogue of S1P receptor agonist that binds to S1PR1 and S1PR3-5 leading to their downregulation (causes transient receptor activation followed by its internalization and degradation), was shown to inhibit tumour vascularization and growth in some cancer models including Lewis lung carcinoma, colon cancer, hepatocellular carcinoma, and prostate cancer [7,8,9,10].

The production of inflammatory mediators in the tumour microenvironment is considered to be a key element in the development of neoplastic disease. It is now established that infectious diseases and chronic inflammation might account for approximately 25% of cancer-causing factors [11]. Many mediators of the inflammatory process interfere with tumour growth and progression. Importantly, some inflammatory cytokines can strongly stimulate tumour growth, invasiveness and metastasis [12]. Bladder cancer (BC) is recognized as a common malignancy, with an incidence of almost 500,000 new cases/year. In addition, it has a high mortality rate, its treatment is expensive and has not improved over a long time [13]. BC accounts for 3% of global cancer diagnoses with about 16,000 deaths only in the USA annually [14]. BC is categorized into two types based on whether the tumour is invading into or beyond the muscularis propia: non-muscle-invasive BC (stage Ta or T1; NMIBC) and muscle-invasive BC (stage T2, T3, or T4; MIBC) [15]. It is believed that about 90% of bladder cancers are confined to the bladder wall layers and of all localized bladder cancers (≤T2), 75% are non-muscle invasive (Tis, Ta, T1) [16]. The remaining 25% of BC cases are affected by MIBC, displaying high histological grade and being responsible for the vast majority of cancer-related deaths [17]. Systemic chemotherapy is the standard initial treatment for unresectable and metastatic forms of BC, however, chemoresistance is becoming a new challenge due to the lack of effective second-line therapies [18]. Despite the fact that standard treatment of NMIBC is associated with good clinical outcomes, new approaches are needed to improve rates of durable cure and bladder preservation for those at the highest risk of progression to MIBC. It has been proven that inflammatory response to *Schistosoma haematobium* infection and other factors triggering chronic inflammation is essential for BC development [19]. On the other hand, it was noticed that bacillus Calmette–Guerin intravesical treatment also causes inflammation, but at the same time ensures cancer-free survival in two-thirds of BC patients [20]. Those findings indicate the dual role of inflammation in BC pathogenesis, that only can be explained by the different spectrum of inflammatory mediators generated in those two conditions. One possible group of molecules that may differ in expression between those two settings are antimicrobial peptides that are released in defence against invading microbes [21]. LL-37 peptide is the only member of the cathelicidin antimicrobial peptide family produced in humans [22]. It has drawn a lot of researchers’ interest in the past decade because it is recognized not only as an important component of the innate immune system but also because it might exert many physiological functions, especially as a stimulator of angiogenesis and cell proliferation [23,24,25]. Additionally, cathelicidin LL-37, regulates cell apoptosis [26,27,28], mechanical properties of epithelial cells, and transepithelial permeability [29]. Since many above-mentioned cellular responses to LL-37 peptide are important in carcinogenesis, an increasing number of experimental works provide evidence indicating the involvement of LL-37 peptide in the development of different types of human cancer [26]. However, available data showed that the LL-37 peptide may act either as a pro-tumorigenic or anti-cancer agent [22,30,31,32,33,34,35]. Indeed, there are essential changes in LL-37 expression in tumour cells comparing to healthy tissue of the same origin [26]. For this reason, the cell signaling pathways that support regulation of the LL-37 peptide synthesis might provide a new targets for pharmacological intervention to prevent cell growth of cancer cells. Interestingly, some recent reports have shown that S1P is able to significantly increase cathelicidin LL-37 production in epithelial cells [36,37,38]. 

In this study, we provide experimental data indicating that the production of LL-37 upon cell stimulation with S1P might stimulate the growth of bladder cancer cells, implementing the possibility of the paracrine interplay between S1P release and LL-37 expression that might be involved in BC development.

## 2. Results

### 2.1. Sphingosine Kinases 1/2 and hCAP-18/LL-37 Are Both Expressed in Human BC Tissues

**Figure 1:**
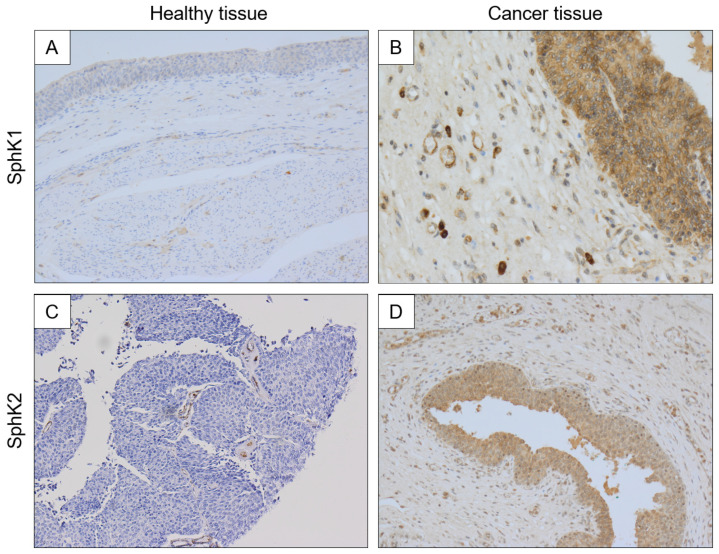
Immunostaining analysis representing SphK1 and SphK2 expression in biopsies collected from normal bladder tissue samples (panels (**A**,**C**)) and human bladder cancer (NMIBC) samples (panels (**B**,**D**)). Magnification 40×.

In the first step of our study to explore the expression pattern of S1P in normal mucosa of the bladder and the neoplastic one, we examined the expression of SphK1 and SphK2 in those tissues using immunohistological staining. As demonstrated in the representative microphotographs (Figure 1), a strong, immunohistochemical (IHC)-positive signal for SphK1/K2 was confirmed in both normal mucosa (Figure 1A,C) and cancer tissues (Figure 1B,D).

**Figure 2:**
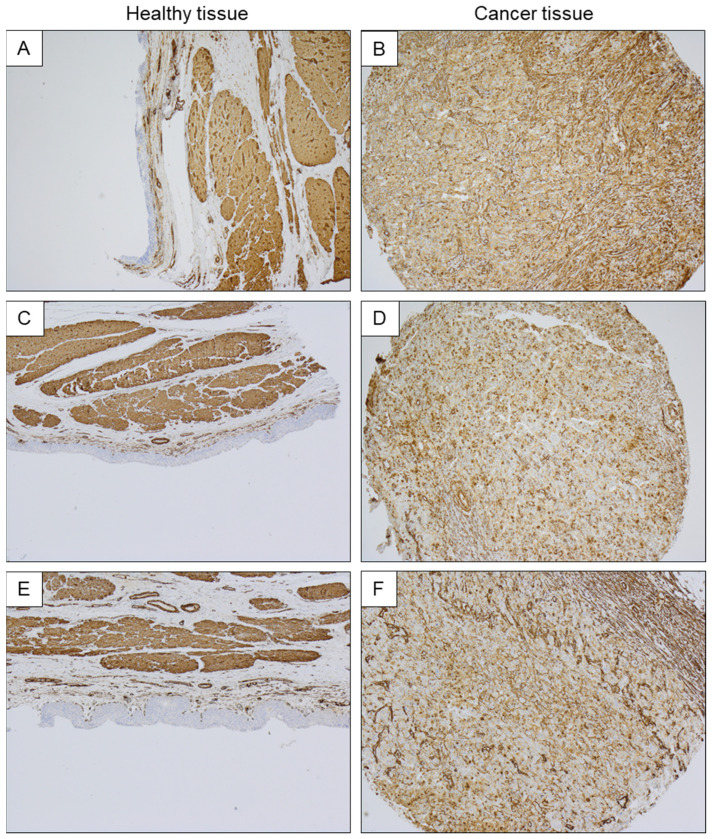
Immunostaining analysis representing hCAP18/LL-37 expression in biopsies collected from normal bladder tissue samples (panels (**A**,**C**,**E**)) and bladder cancer tissue (NMIBC) samples (panels (**B**,**D**,**F**)). Magnification 40×.

Simultaneously, we detected that hCAP-18/LL-37 expression varied between healthy and cancerous tissues. Accordingly, the samples collected from healthy bladder mucosa revealed a negative IHC test for hCAP-18/LL-37 peptide (Figure 2A,C,E), while in all tested samples collected from cancer tissue, a positive mucosa IHC signal for hCAP-18/LL-37 in cancer cells was recognized (Figure 2B,D,F).

**Figure 3:**
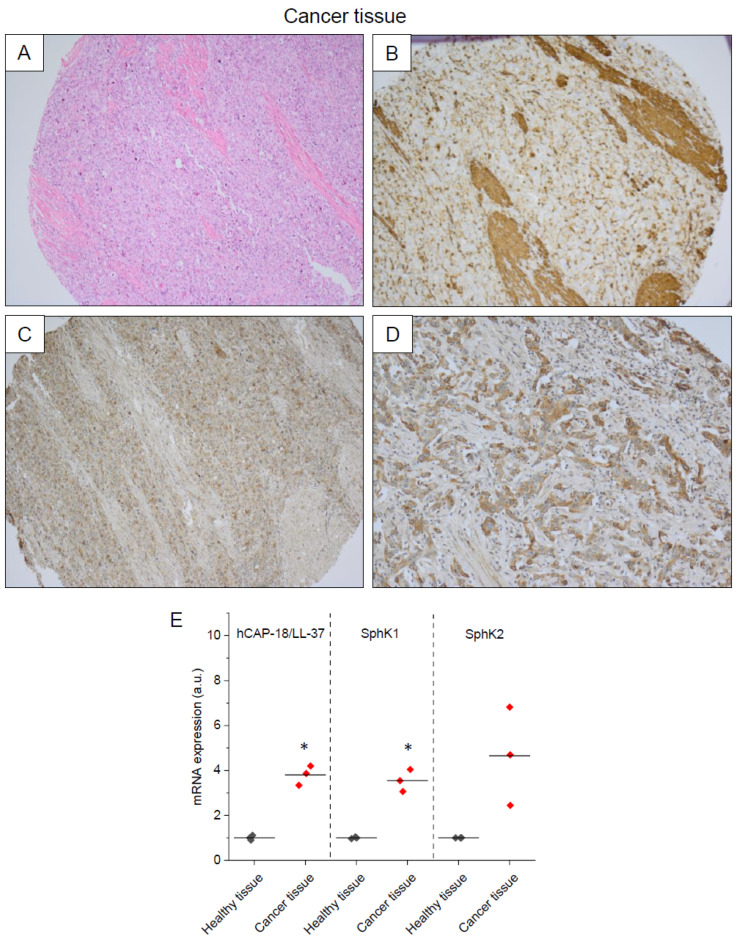
Immunostaining analysis representing hCAP18/LL-37 (panel (**B**)), SphK1 (panel (**C**)) and SphK2 (panel (**D**)) expression in the in biopsies collected from human bladder cancer (NMIBC) samples (panel (**A**)). Expression of hCAP18/LL-37, SphK1 and SphK2 mRNA in healthy and cancer tissues is presented in panel (**E**). Magnification 40×. * indicates statistical significance (*p* < 0.05) when compared to healthy tissue.

Notably, a co-expression of SphK1 and hCAP-18/LL-37 in bladder-cancer-affected tissue was also confirmed (Figure 3A–D). The above observations were also confirmed using qRT-PCR analysis; in cancer samples, an increased expression of hCAP-18/LL-37, SphK1 and SphK2 mRNA when compared to healthy tissues was detected (Figure 3E).

Collectively, those data strongly suggest that both SphK1 and hCAP-18/LL-37 might be recognized as molecules affecting bladder cancer initiation and progression. Nevertheless, it should be noted that we only investigated the material from a small group of patients (*n* = 20), which is a limitation of our study. Additionally, in the case of SphK1 staining, in two cases the staining was negative both in cancer and closely connected healthy tissue.

### 2.2. S1P Promotes the Proliferation of Human Bladder T24 Cells and This Effect Is Restricted by Pre-Treatment with FTY720P

**Figure 4:**
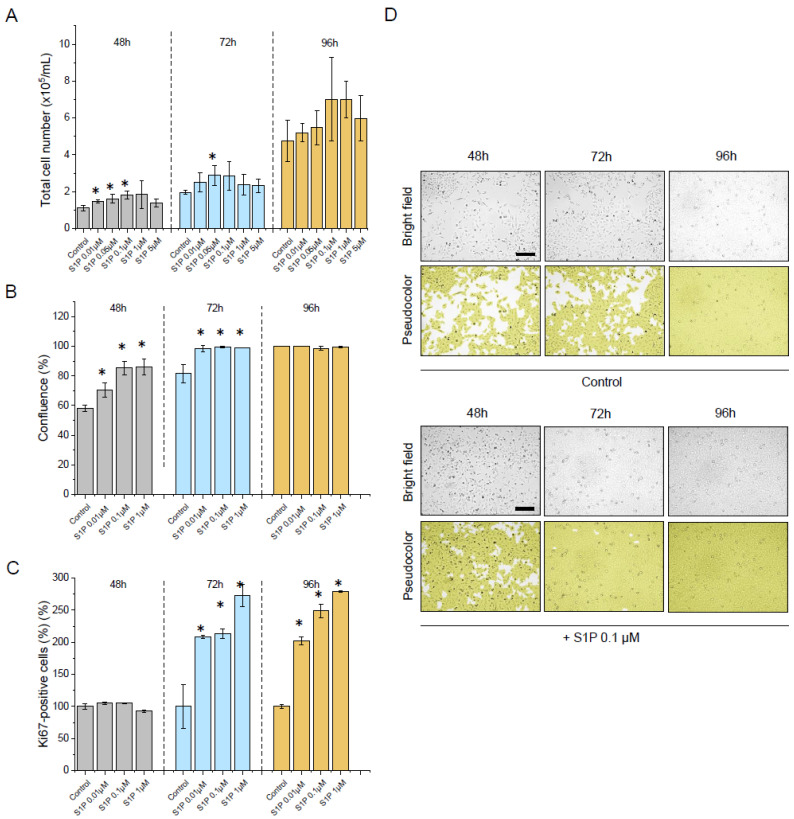
The proliferation of human bladder T24 (ATCC^®^ HTB-4™) cells subjected to S1P. An increase in the total number of cells incubated in the presence of S1P at concentrations ranging from 0.1 µM to 5 µM for 48 (grey bars), 72 (blue bars), and 96 h (brown bars), the confluence of respective cell populations and number of Ki67-positive cells when compared to untreated control are presented in panels (**A**–**C**), respectively. In panel (**D**) representative microphotographs of the S1P-treated cellular population upon culturing for 48, 72, and 96 h are shown. Alterations in cellular confluence are presented using bright field (**upper** row) and pseudocolour (**lower** row). Scale bar equals 10 µm. For panels (**A**–**C**) results are presented as mean ± SD from 3 repetitions. For panel (**D**) the results of one representative experiment are shown. * indicates statistical significance (*p* < 0.05) when compared to untreated control cells.

Inflammation and cellular stress are able to induce the generation of sphingolipids with anti-apoptotic functions [39]. It was proven that dysregulation of the S1P-dependent pathway contributes to the development and progression of cancer, including bladder cancer, affecting the poor survival in this group of patients [5,40]. For this reason, following the experiments presented above, a cell culture model was employed to quantify the effect of S1P on human bladder cancer cell proliferation after 48, 72, and 96 h of incubation (Figure 4). As demonstrated, a remarkable enhancement of the total number of cells grown in the presence of S1P (Figure 4A), as well as an increase in cellular confluence and number of Ki67-positive cells (Figure 4B–D) was noted, confirming the stimulatory effect of this compound on cellular division up to 1 µM of S1P. 

**Figure 5:**
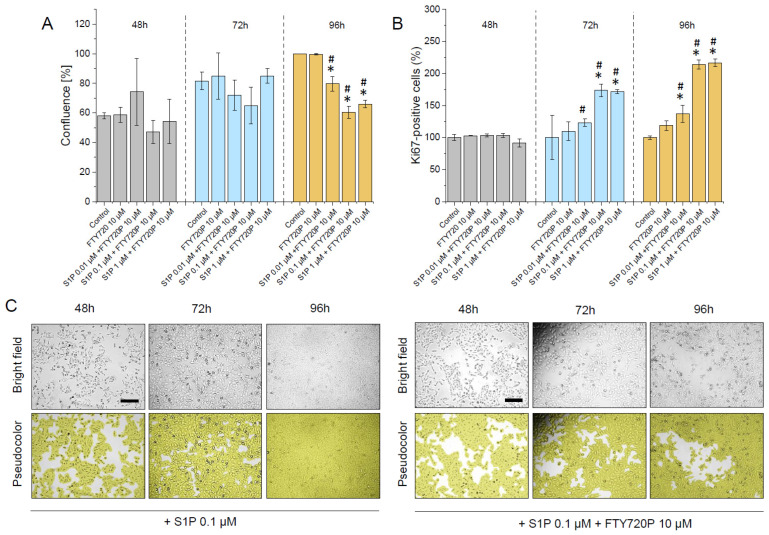
Restriction of human bladder T24 (ATCC^®^ HTB-4™) cells proliferation upon FTY720P addition. The confluence of S1P-treated human bladder T24 (ATCC^®^ HTB-4™) cell population, when preincubated with FTY720P (10 µM), as well as number of Ki67-positive cells is presented in panels (**A**,**B**). Representative microphotographs of the cellular population treated with S1P (0.1 µM) or pre-treated with FTY720P (10 µM) and then cultured in the presence of S1P (0.1 µM) for 48 (grey bars), 72 (blue bars) and 96 h (brown bars) are presented in panel C. Alterations in cellular confluence are presented using bright field (upper row) and pseudocolour (lower row). Scale bar equals 10 µm. For panels (**A**,**B**) results are presented as mean ± SD from 3 repetitions. For panel (**C**) the results of one representative experiment are shown. * and # indicate statistical significance (*p* < 0.05) when compared to untreated control cells and S1P-treated cells, respectively.

Simultaneously, as shown in Figure 5, the effect of S1P on human bladder cells was strongly restricted by the addition of FTY720P which is known to induce long-term downregulation of S1PR receptors (precisely S1PR1, S1PR3, SP1R4 and S1PR5). These effects were shown both after 72 and 96 h.

### 2.3. Release of Ll-37 from Human Bladder Cancer Cells Is Triggered by S1P

**Figure 6:**
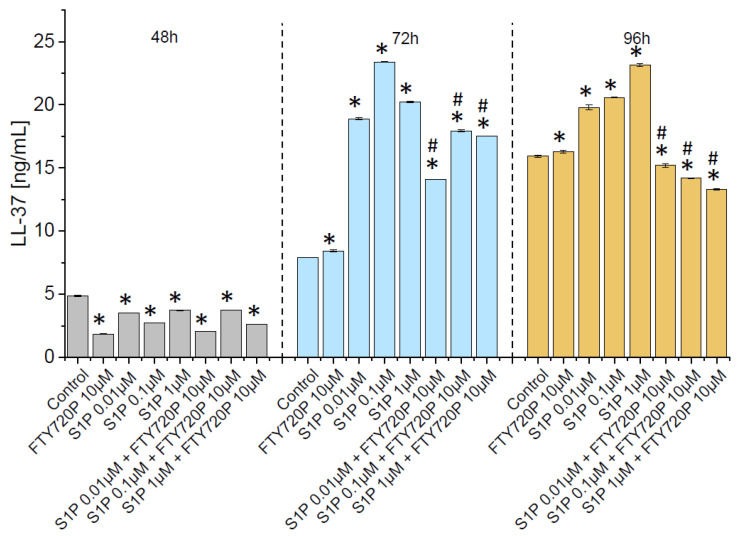
S1P-stimulated induction of LL-37 production in urinary bladder cells. Effect of S1P and FTY720P on LL-37 release from stimulated T24 (ATCC^®^ HTB-4™) cells is shown in panel. Cells were incubated with indicated agents for 24 (grey bars), 48 (blue bars), and 96 h (brown bars). Results are presented as mean ± SD from 2 duplicates. * and # indicate statistical significance (*p* < 0.05) when compared to untreated control cells and S1P-treated cells, respectively.

It is established that in urothelial cells, renal epithelial cells, and neutrophils, LL-37 is constitutively expressed at low levels [41], but its production might be considerably increased in the response to microbial assault or inflammatory factors [42]. Some studies demonstrate the possible impact of S1P on LL-37 production. For instance, Park et al. reported that S1P mediates endoplasmic reticulum (ER) stress-induced generation of cathelicidin antimicrobial peptide (CAMP), suggesting that S1P by modulation of antimicrobial peptides production might comprise a novel regulatory mechanism of immune machinery [36]. Since the impact of this effect on the release of LL-37 in bladder cancer is obscure, we decided to explore whether S1P might promote the release of cathelicidin from stimulated bladder cancer cells. As shown in Figure 6, subjection of human bladder cancer cells with S1P resulted in an increase of hCAP-18/LL-37 release in the cell culture model. The phenomenon was particularly prominent after 96 h of incubation. It is worth noticing that increased release of LL-37 was observed after incubation with increasing doses of S1P. Importantly, the opposite trend was found when the effect of S1P-mediated signalling pathways was blocked by the addition of FTY720P (Figure 6). Notably, a similar tendency was noted in other LL-37-stimulated cancer cells, including breast, lung and ovarian cells confirming the importance of S1P-LL-37 interactions (Supplementary Figure S1).

### 2.4. Exogenous Ll-37 Peptide Exerts Stimulatory Effect on Proliferation of Human Bladder Cancer Cells

**Figure 7:**
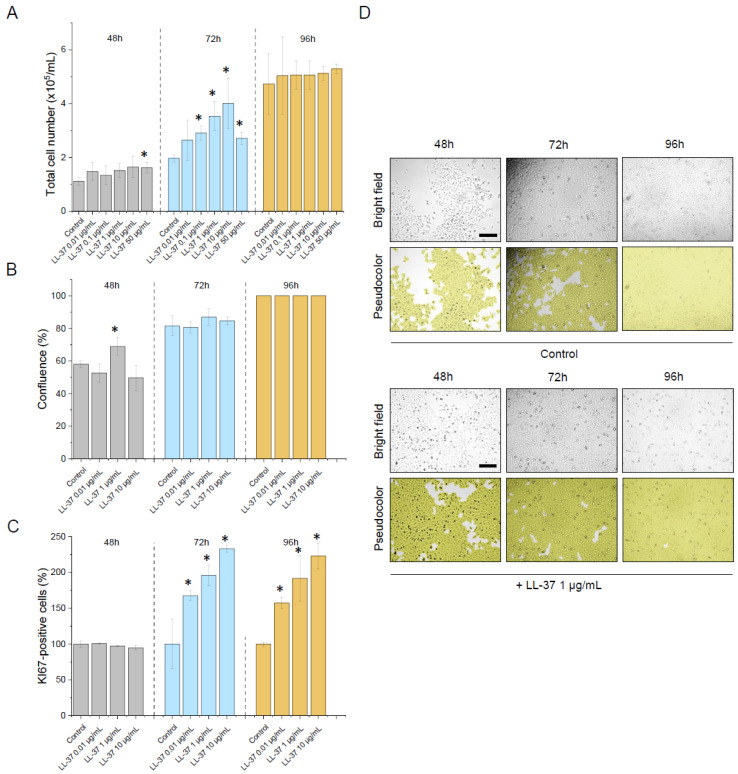
Impact of LL-37 peptide on human bladder T24 (ATCC^®^ HTB-4™) cells’ proliferation. An increase in the total number of cells incubated in the presence of LL-37 at concentrations ranging from 0.01 µg/mL to 50 µg/mL for 48 (grey bars), 72 (blue bars), and 96 h (brown bars), the confluence of respective cell populations and number of Ki67-positive cells are presented in panels (**A**–**C**), respectively. In panel (**D**) representative microphotographs of the LL-37-treated cellular population upon culturing for 48, 72, and 96 h are shown. Alterations in cellular confluence are presented using bright field (**upper** row) and pseudocolour (**lower** row). Scale bar equals 10 µm. For panels (**A**–**C**) results are presented as mean ± SD from 3 repetitions. For panel (**D**) the results of one representative experiment are shown. * indicate statistical significance (*p* < 0.05) when compared to untreated control cells.

LL37, being recognized as an important element of the innate defence in the urinary tract, is also well known as a stimulator of cell proliferation and a promoter of growth of many tumours [26,43]. To assess whether indeed LL-37 displayed a pro-tumorigenic effect in our experimental settings, non-stimulated human bladder cells were incubated in the presence of LL-37 at concentrations ranging from 0.01 µg/mL to 50 µg/mL up to 96 h (Figure 7). Accordingly, we have noticed a significant and dose-dependent increase in cell proliferation after 72 h of incubation, which was quantified by the enhancement of total cell number, increased cell population confluence and number of proliferating, Ki67-positive cells.

## 3. Discussion

The nature of the specific factors triggering the development of bladder cancer in vivo remained unclear. Compelling evidence demonstrates that a protracted inflammatory process, which originally is a self-limiting host defence mechanism against biological, chemical, and physical agents, could lead to cellular damage associated with prolongate regeneration causing cancer development [44]. In accordance with this statement, it was established that besides smoking habits and occupational exposure to aromatic amines, chronic inflammation should be recognized as a leading risk factor for bladder cancer [19]. Although the molecular mechanism supporting this process is not fully understood, patients with chronic urinary tract infections, chronic use of urinary catheters, and bladder stones are at increased risk of bladder cancer [45].

The role of sphingolipids in cancer development and progression is multidirectional, complex, and not well understood. It has been shown that S1P is able to influence inflammatory responses and carcinogenesis in different tissues. Moreover, data are showing that the S1P signalling pathway could play an important role as a therapeutic target for the prevention and treatment of inflammation-triggered cancers [46]. Our preliminary immunohistochemical analysis conducted on human bladder cancer samples has shown a positive IHC mucosa signal for LL-37 and SphK1 in cancer tissues. These results motivated us to turn our research to cell culture models to understand the role of LL-37 and S1P in BC. Numerous previously published studies revealed that LL-37 had a huge range of pleiotropic properties [24,31]. Moreover, its actions are sometimes unexpected [47,48]. For the purpose of the current study, it is important to underline that LL-37 can regulate some aspects of cell behaviour that are directly linked with cancer development such as cell proliferation, epithelial cell migration, and wound closure, in that way regulating tissue homeostasis and regenerative processes [26,27]. Moreover, it has been suggested that cathelicidin could play an important role in the regulation of apoptosis since its angiogenic effects have been documented [49]. The loss of the balance between cell proliferation and cell death makes a direct pathway to tumour development [50], therefore the LL-37 regulation of apoptosis may be involved in malignant tumour growth. As shown in previous studies, LL-37-induced apoptosis is recognized as its antitumour activity in colon cancer and some hematologic malignancies [28,51,52]. Interestingly, LL-37 can display a variable impact on malignancy growth, depending on the tissue type from which cancer cells come, considering tissue embryonic background. In fact, in different types of cancer, various level of expression of LL-37 was reported. In more detail, in ovarian, lung, breast tumours, and malignant melanoma an increased expression was found [53,54,55,56,57]. In contrast, LL-37 production in colon and gastric cancer cells was diminished [28,58]. So far, the mechanisms responsible for the pro-tumorigenic or anti-cancer effect of LL-37 are not fully elucidated. However, based on the observations mentioned above, it has been suggested that the actions of this peptide are strongly dependent on the ability of human cathelicidin to interact with a spectrum of membrane receptors whose expression varies on different cancer cells and for this reason, its effects are tissue-specific [26] or may be dependent on other factors. Our observations indicate that S1P might act as a paracrine mediator that links pro-inflammatory response in tissue (in our case bladder’s mucosae) with tissue proliferation, via up-regulation of hCAP-18/LL-37 expression and release. This motion is justified by the observation of SphK staining in tissue samples obtained from bladder cancer and a cell culture system in which bladder cancer can release hCAP18/LL-37 in response to the addition of S1P. Indeed, S1P, a product of sphingosine kinases (SphK1 and 2), was identified as a mediator of pro-inflammatory events [59] and some previous investigations have also pointed out the role of sphingolipids in malignancy [3]. In addition, it has been shown here that FTY720P, an S1P receptor inhibitor, was able to suppress the growth and malignancy of cancer in several experimental models [3]. It is in accordance with previous observations, where the sphingolipid axis responsible for anti-apoptotic and growth-promoting actions was necessary for malignant transformation during inflammation and injury in various tissues [46,60]. The role of the SphK1/S1P network was also recognized as a crucial one in gastrointestinal tract oncogenesis, where inflammation was considered the most important initiating factor [61]. S1P, acting as an oncopromotor, is involved in tumour growth and invasiveness; some of these effects have been attributed to its ability to regulate the expression of many growth factors, including epidermal growth factor (EGF). As shown in previous studies, S1P either directly transactivated EGF receptors (EGFR) in gastric cancer cells or was responsible for upregulating EGFR in lung or breast cancer cells, contributing to tumour progression [62,63,64]. The biological actions of LL-37 that were implicated in some types of malignancies were also mediated by the activation of EGFR [57]. We speculate that the ability of S1P to stimulate the release of LL-37 from bladder cells, which could serve as a direct agonist of EGFR, coupled with the ability of S1P to directly upregulate this receptor, could potentiate the carcinogenic effect of S1P on the inflammatory side. This functional link between S1P and EGF signalling pathways has already been described in human glioblastoma multiforme cell lines [65]. As suggested in these studies, a possible association between the S1P and EGFR signalling pathways was associated with greater tumour invasiveness and poor prognosis.

## 4. Materials and Methods

### 4.1. Material

Samples for immunohistochemical analysis were obtained from twenty patients (15 men and 5 women, aged 55 ± 8) with a non-muscle-invasive form of bladder cancer (NMIBC) taken after surgical procedures. Human urinary bladder cancer T24 (ATCC^®^ HTB-4™) cells and McCoy’s 5A Medium Modified were purchased from American Type Culture Collection (ATCC; Manassas, VA, USA). S1P and LL-37 peptide were from Sigma Aldrich (Saint Louis, MI, USA) and Lipopharm.pl (Gdańsk, Polska), respectively. The S1P analog, fingolimod in its bioactive form (FTY720P) was from Sigma Aldrich. Muse^®^ Count & Viability Kit was ordered from Luminex Corporate (Austin, TX, USA). The human cathelicidin LL-37 (hCAP-18/LL-37) ELISA kit was from Hycult Biotech (Uden, The Netherlands). Antibody against hCAP-18/LL-37 peptide was from Santa Cruz Biotechnology, (Dallas, TX, USA).

### 4.2. SphK1 and SphK2 Immunohistochemical Analysis

Antigen retrieval for pSphK1 and pSphK2 was performed by microwaving the slides under pressure in a citrate buffer for 10 min (pH 9.0). Endogenous peroxidase was blocked using 0.3% hydrogen peroxide for 20 min. After blocking the nonspecific background, the sections were incubated overnight with the primary antibody (pSphK1 or pSphK2 polyclonal antibody; 1:100 dilution; Invitrogen ThermoFisher Scientific, Waltham, MA, USA) at 4 °C. Then, the sections were incubated with biotinylated rabbit anti-mouse streptavidin-peroxidase complex for 10 min. Diaminobenzidine was used as the chromogen, and the sections were counterstained with hematoxylin. Normal mouse immunoglobulin was substituted as the primary antibodies in the negative control. The vascular and lymphatic endothelial cells of all vessels reacted with the antibody against pSphK1 or pSphK2. As a result, the pSphK1 or pSphK2 staining intensity of endothelial cells is considered strong staining, and semi-quantitative evaluation of the pSphK1 or pSphK2 staining intensity of bladder cancer cells was registered as follows: 0 (negative), 1 (weak), 2 (moderate), or 3 (strong), based on comparison with endothelial cell staining. The staining score 0 and 1 were considered to be pSphK1 or pSphK2 negative and 2 and 3 were considered to be pSphK1 or pSphK2 positive, respectively.

### 4.3. hCAP-18 Immunohistochemical Analysis

Four-micron freshly cut sections (<2 weeks) of formalin-fixed, paraffin-embedded (FFPE) tissue of 3 cases were dried and melted in a 62 °C oven for 20 min. Subsequently, they were stained with mouse monoclonal LL-37 antibody (1/500 titer) according to the manufacturer’s protocol. In short, staining was performed on the Ventana BenchMark XT (Ventana Medical Systems Inc., Oro Valley, AZ, USA). The staining protocol included online deparaffinization, HIER (Heat Induced Epitope Retrieval) with Ventana Cell Conditioning 1 for 32 min, and primary antibody incubation for 20 min at 31 °C. Antigen-antibody reactions were visualized using the Ventana OptiViewTM Amplification kit, followed by Ventana OptiViewTM Universal DAB Detection Kit (Optiview HRP Multimer 8 min, Optiview Amplifier H_2_O_2_/Amplifier 4 min, Optiview Amplifier Multimer 4 min, Optiview H_2_O_2_/DAB 8 min, Optiview Copper 4 min). Counterstaining was obtained using Ventana Hematoxylin II for 8 min followed by bluing reagent for 4 min. Finally, all slides were removed from the stainer, dehydrated, and cover-slipped for microscopic examination. Positive control included a known hCAP-18/LL-37 positive human spleen tissue. Cases were scored as positive (“IHC positive”), negative (“IHC negative”), and “negative-nonspecific” (“IHC negative/nonspecific”). Only tumour cells showing non-ambiguous cytoplasmic and membrane staining for hCAP-18/LL-37 immunostaining were scored as positive (“IHC positive”). The faint, weak granular stain was noted in a few cases and considered nonspecific. These cases were scored as “IHC negative/nonspecific” for tracking purposes. IHC slides were scored first by a primary reviewer and later by an additional independent reviewer, all four blind to the molecular results. The primary reviewer (JK) was the one most intimately involved wWe changed this to subscript, please confirm the proposed changesith the development of the immunostaining, who had gained the most experience through the process, and was most familiar with variations in stain intensity and distribution not only among PTC cases but also with other positive and negative controls. Due to the level of expertise in reading the hCAP-18/LL-37 immunostaining and the sequence of events, the primary reviewer was used as the “gold standard” to which the results of the hCAP-18/LL-37 molecular analysis were compared (see below). The two reviewers were compared to each other in regards to the scoring of hCAP-18/LL-37 immunohistochemistry.

### 4.4. qRT-PCR Analysis

Formalin-fixed paraffin-embedded tissue sections were deparaffinized by submerging twice in xylene for 30 min each and hydrated with an alcohol gradient (absolute, 70%, and 50%) for 2 min. Then sections were rinsed with nuclease-free water and transferred to 2 mL plastic tubes. Total RNA was isolated using the High Pure FFPET RNA Isolation Kit (Roche, Basel, Switzerland) according to the protocol provided by the manufacturer. The concentration and purity of isolated RNA were evaluated using a Nanodrop 2000 (Thermofisher, Waltham, MA, USA). cDNA was synthesized using the iScript™ cDNA Synthesis Kit (Bio-rad, Hercules, CA, USA). qRT-PCR was performed with 50 ng of cDNA in 15 μL of QuantiTect^®^ SYBR^®^ Green PCR (Qiagen, Hilden, Germany) on the PikoReal™ Real-Time PCR System (Thermofisher, Waltham, MA, USA). GAPDH was used as an internal control. Gene expression levels were analyzed using the 2^−ΔΔCt^ formula. The sequence of the primers used in the study is presented in Table 1.

### 4.5. Cell Culture

T24 (ATCC^®^ HTB-4™) cells were cultured in McCoy’s 5A Medium Modified with 10% fetal bovine serum (FBS), 2 mM glutamine, and 1% of antibiotics (100 units penicillin, 100 µg streptomycin and 0.25 μg amphotericin B per mL) at 37 °C and 5% CO_2_. For experiments, cells were seeded at a density of 2.5 × 10^4^ cells/well in 24-well plates and cultured either with S1P (at a concentration range from 0.01 µM to 5 µM) or LL-37 (concentration range from 0.01 µg/mL to 50 µg/mL) up to 96 h. For experiments aiming to block S1P-mediated signalling pathways, cells were pre-treated with FTY720P at a concentration of 10 µM for 1 h before incubation either with S1P or LL-37.

### 4.6. Cell Counting and Confluence Assessment

At indicated time points, both control and compound-treated cells were examined in the aspect of total cell number, a confluence of grown population, and morphology of cells. The counting of cells was performed using Muse^®^ Count & Viability Kit according to the manufacturer’s guidelines. A confluence of cells on the surface of the cell culture plate was inspected using light microscopy at 40× magnification and calculated as a mean ± SD from 5 different view fields using the automatic cell imaging system Juli™ Br (NanoEntek, Seoul, Korea).

### 4.7. Examination of LL-37 Production in T24 (ATCC^®^ HTB-4™) Cells

To explore whether stimulation of cells with S1P might affect the production of LL-37 peptide, urinary bladder cells were treated either with S1P alone or FTY720P/S1P and cell culture supernatant was collected upon 48 h, 72 h, and 96 h incubation. The concentration of LL-37 in the cell culture medium was measured using a human cathelicidin LL-37 ELISA kit, according to the manufacturer’s guidelines.

### 4.8. Statistical Analysis

Results are presented as mean ± SD from three repetitions. Microphotographs from the histopathological analysis are representative of 20 samples, tested for each group. The significance of differences between tested samples and control wells was determined using the two-tailed Student’s *t*-test. Statistical analyses were performed using OriginPro 2020 (OriginLab Corporation, Northampton, MA, USA). *p* < 0.05 was considered to be statistically significant.

## 5. Conclusions

In summary, in our experiments, we have demonstrated that LL-37 markedly and dose-dependently increased the proliferation of human bladder cells. Secondly, we have proven that S1P can stimulate the expression/release of hCAP-18/LL-37 from stimulated human bladder cells. When cells were pre-treated with FTY720P, the effect of S1P on LL-37 release was inhibited. Therefore, the present study suggests that S1P can increase the proliferation of bladder cells through two potential mechanisms: directly acting on its cellular receptors and indirectly—by influencing the release of hCAP-18/LL-37. These preliminary data are encouraging for further research on the impact of S1P and LL-37 on the development of urinary bladder malignancies. 

## Figures and Tables

**Table 1:** Primers used for quantitative RT-PCR.

Gene	Primer Direction	Sequence 5′->3′ (DNA)	Annealing Temperature (°C)
hCAP-18/LL-37	forward	GAAGACCCAAAGGAATGGCC	53.8
	reverse	CAGAGCCCAGAAGCCTGAGC	57.9
SPHK1	forward	GCTGGCAGCTTCCTTGAACCAT	56.7
	reverse	GTGTGCAGAGACAGCAGGTTCA	56.7
SPHK2	forward	GAGGAAGCTGTGAAGATGCCTG	56.7
	reverse	GAGCAGTTGAGCAACAGGTCGA	56.7
GAPDH	forward	GTCTCCTCTGACTTCAACAGCG	56.7
	reverse	ACCACCCTGTTGCTGTAGCCAA	56.7

## Data Availability

The data presented in this study are available on request from the corresponding author.

## Supplementary Materials

The following supporting information can be downloaded at: https://www.mdpi.com/article/10.3390/ijms23137443/s1.
