# Supplementary material for: Sphingosine-1-Phosphate-Triggered Expression of Cathelicidin LL-37 Promotes the Growth of Human Bladder Cancer Cells
Source: Int J Mol Sci. 2022 Jul 4;23(13):7443. doi: 10.3390/ijms23137443 (PMC9267432; doi:10.3390/ijms23137443)
Supplement: Supplementary file 1 [file ijms-23-07443-s001.zip › ijms-1711937-supplementary.pdf]

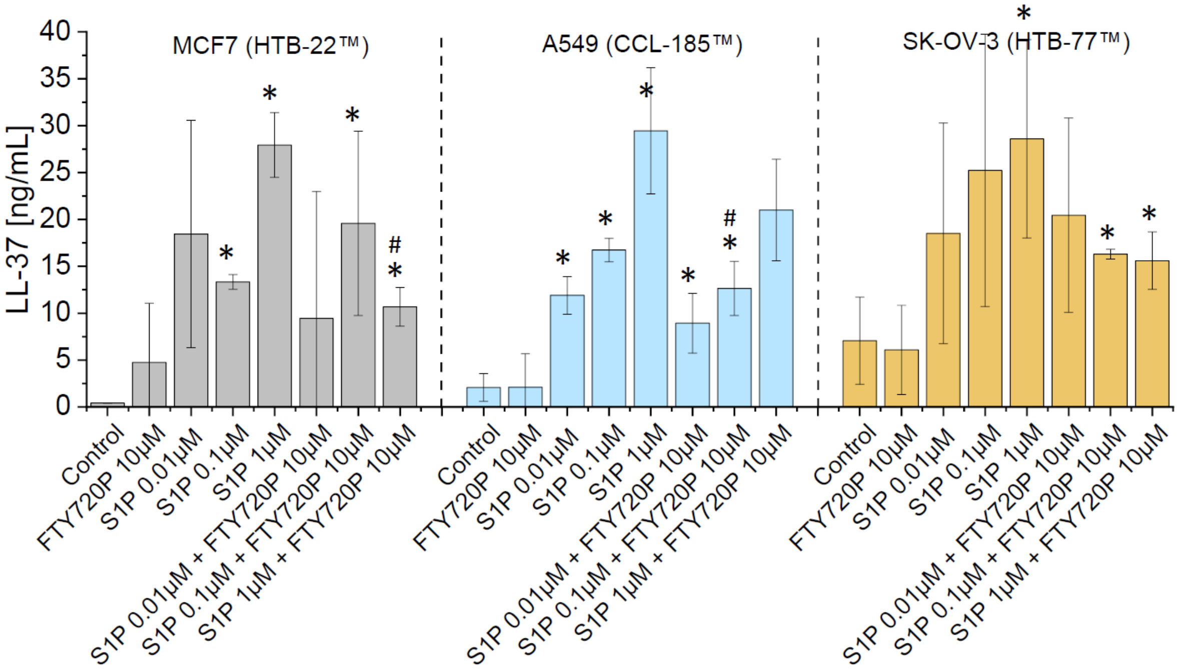

Supplementary Figure S1. S1P-stimulated induction of LL-37 production in breast, lung, and ovarian cancer cells. Effect of S1P and FTY720P on LL-37 release from stimulated MCF7 (HTB-22™), A549 (CCL-185™), and SK-OV-3 (HTB-77™) cells is presented using grey, blue and brown bars, respectively. Cells were incubated with indicated agents for 72 hours before ELISA measurements. Results are presented as mean ± SD from 2 duplicates. * and # indicate statistical significance ($p<0.05$) when compared to untreated control cells and S1P-treated cells, respectively
